# Genome Sequences of Diverse Human Cytomegalovirus Strains with Utility in Drug Screening and Vaccine Evaluation

**DOI:** 10.1128/genomeA.01433-16

**Published:** 2017-01-19

**Authors:** Kathleen Corcoran, Carly J. Sherrod, Ellen F. Perkowski, Jordan Texier, Fengsheng Li, I-Ming Wang, Michael McVoy, Tong-Ming Fu, Dirk P. Dittmer

**Affiliations:** a Department of Microbiology and Immunology, Lineberger Comprehensive Cancer Center Program in Global Oncology, Center for AIDS Research (CfAR), School of Medicine, University of North Carolina at Chapel Hill, Chapel Hill, North Carolina, USA; b MRL, Merck & Co., Inc., Kenilworth, New Jersey, USA; c Department of Pediatrics, Virginia Commonwealth University School of Medicine, Richmond, Virginia, USA

## Abstract

Cytomegalovirus displays genetic heterogeneity, which has implications for antiviral and vaccine development. Many studies have focused on laboratory isolates that have been extensively adapted for growth on fibroblasts. Here, we report whole-genome sequences for 10 human cytomegalovirus (HCMV) strains that readily grow on ARPE-19 human retinal pigment epithelial cells.

## GENOME ANNOUNCEMENT

Human cytomegalovirus (HCMV) is a herpesvirus in the subfamily *Betaherpesvirinae*, which, like all herpesviruses, establishes lifelong latent infections. Primary infection is usually mild to asymptomatic; however, for immunocompromised individuals, such as AIDS patients, transplant recipients, and developing fetuses, HCMV infection can have profound implications. HCMV isolates display genetic heterogeneity (1–3). Thus, genomic sequence data from a comprehensive collection of different isolates will facilitate vaccine and antiviral drug development.

Ten HCMV strains were collected from a diverse set of sources. VHL/E was isolated from a duodenal biopsy specimen from a bone marrow transplant patient, propagated, and passaged in human umbilical vein endothelial cells (4, 5). VR3908 and VR7863 were isolated from urine samples from congenitally infected neonates and cultured on endothelial and epithelial cells (6). SUB-22 and SUB-24 were isolated from urine samples from congenitally infected neonates (S. Adler, unpublished data). VR5201, VR5235, and VR5022 were recovered from blood samples from solid organ transplant patients (6). NR was isolated from a serum sample from a kidney transplant patient and cloned into a bacterial artificial chromosome (H. Zhu, unpublished data). TB40/E was isolated from a throat swab from a bone marrow transplant patient, culture adapted, cloned into a bacterial artificial chromosome, and later modified by the addition of a green fluorescent protein (GFP) cassette (7, 8). Prior to sequencing, all strains were passaged three to five times on ARPE-19 human retinal pigment epithelial cells (9). Peak titers of culture supernatants ranged from 10^3^ to 10^5^ PFU/ml. Epithelial entry efficiency, assessed as relative infectivity on ARPE-19 cells versus MRC-5 fibroblasts, ranged from 10 to 80%.

The VHL/E genome was assembled from 5,234,206 Illumina read pairs (*n* = 90). A *de novo* assembly was first generated using CLC Assembler. The Illumina reads were then read normalized and error corrected using BBMap tools (version 35.82), yielding 937,689 pairs with a mean distance of 454 bp that remapped to the *de novo* draft assembly. A total of 1,804 incorrectly oriented pairs and 3,953 distant pairs were excluded. The final consensus was constructed requiring >50% identity at each position, with a mean ± standard deviation (SD) coverage of 570.5× ± 64.3×.

The nine remaining genomes were sequenced on a 454 GS Junior sequencer (Roche), according to the manufacturer’s protocol. Reads were trimmed and filtered for quality and read length using CLC Genomics Workbench version 9.0 (CLC bio). Filtered reads were used for *de novo* assembly, and contigs greater than 10 kbp were used to determine suitable reference sequences via NCBI nucleotide BLAST. Original trimmed reads were mapped to that reference (10). Reads that could nonspecifically map to a single position were ignored, and consensus sequences were generated with at least 25× coverage. Known repeat regions, with less than 25× coverage, were filled from corresponding references.

These genome sequences provide a diverse collection of HCMV strains that retain epitheliotropism. This collection of sequences, representing strains from diverse sources and geographical regions, should facilitate studies to evaluate antiviral sensitivities or the impact of antigenic polymorphisms on vaccine efficacy.

### Accession number(s).

The assembled sequences for HCMV are deposited in GenBank with the following accession numbers: NR, KX544831; SUB_24, KX544832; VR3908, KX544833; SUB_22, KX544834; VR5022, KX544835; VR5201, KX544836; VR5235, KX544837; VR7863, KX544838; TB40-E_UNC, KX544839; VHL/E_Merck_UNC, KX544841.
